# Comparative Efficacy and Safety of Stillen^®^ and Rebamipide in Patients with Acute or Chronic Gastritis: A Systematic Review and Network Meta-Analysis of Randomized Controlled Trials

**DOI:** 10.3390/jcm14176209

**Published:** 2025-09-02

**Authors:** Hyejoo Lee, Na Rae Lim, Seonwoo Kim, Hyun Cho, Woo Chul Chung

**Affiliations:** 1 Academic Research Service Headquarters, LSK Global PS, Seoul 04535, Republic of Korea; lee.hyejoo@lskglobal.com (H.L.); kim.seonwoo@lskglobal.com (S.K.); cho.hyun@lskglobal.com (H.C.); 2 Department of Internal Medicine, St. Vincent Hospital, College of Medicine, The Catholic University of Korea, Seoul 06591, Republic of Korea

**Keywords:** gastritis, Stillen^®^, rebamipide, network meta-analysis, non-inferiority

## Abstract

**Background**: Erosive gastritis has various causes, and severe damage to the mucosa can cause symptoms such as indigestion, abdominal pain, nausea, and vomiting. The condition is defined by visible erosions that emerge as discrete defects in the gastric mucosa. Stillen^®^ is a natural mucosal-protective agent derived from *Artemisia asiatica*, but its comparative efficacy versus rebamipide remains unclear. This systematic review and network meta-analysis evaluated whether Stillen^®^ leads to non-inferior endoscopic improvement outcomes compared to rebamipide in patients with acute or chronic gastritis. **Methods**: Databases including PubMed, EMBASE, Cochrane Library, RISS, KoreaMed, ClinicalTrials.gov, and ICTRP were searched through 15 August 2025. Eligible studies were randomized controlled trials (RCTs) comparing Stillen^®^ with rebamipide or other mucosal-protective agents in gastritis. Two reviewers independently screened studies, extracted data, and evaluated the risk of bias. The primary outcome was the improvement rate (≥50% erosion reduction); the secondary outcome was the cure rate (complete erosion resolution) and safety outcomes. **Results:** A total of 11 RCTs met the inclusion criteria, with 9 contributing to the primary network analysis. Stillen^®^ was non-inferior to rebamipide in terms of the improvement rate at 2 weeks (OR: 1.11, 95% CI: (0.88, 1.39) in FAS) and in pooled analysis across 2, 3, and 4 weeks. Cure rates showed no significant differences between the two agents. The safety outcomes were comparable, with no significant differences. Most studies were assessed as having a low risk of bias, and the certainty of evidence was rated as high for most efficacy outcomes. **Conclusions:** The network meta-analysis suggests that Stillen^®^ is non-inferior to rebamipide in improving erosions in patients with acute or chronic gastritis.

## 1. Introduction

Erosive gastritis is a prevalent gastrointestinal disorder characterized by endoscopically visible mucosal erosions, which is caused by multiple etiologies, and severe mucosal damage may lead to symptoms such as indigestion, abdominal pain, nausea, and vomiting [1]. The pathogenesis of erosive gastritis involves several factors, such as *Helicobacter pylori* infection, which increases gastric acid secretion and triggers inflammation [2,3], and the use of nonsteroidal anti-inflammatory drugs (NSAIDs), which compromise mucosal defenses. NSAIDs remain widely used for managing pain and inflammation, and their associated gastrointestinal side effects, such as gastric mucosal damage, increase the clinical burden [4,5].

While the etiology of erosive gastritis can be multifactorial, current major clinical practice guidelines, including those from the American College of Gastroenterology (ACG), and the Asian Pacific Association of Gastroenterology (APAGE), primarily focus on treating specific underlying causes, such as *Helicobacter pylori* infection, the prevention of ulcers in patients using NSAIDs or acid-suppressive agents such as proton pump inhibitors (PPIs) and histamine H2 receptor antagonists (H2RAs) for acid-related disorders such as gastroesophageal reflux disease (GERD) [6,7,8]. Explicit and independent guidance on the management of gastritis itself—particularly regarding the selection and comparative efficacy of mucosal protective agents—is limited.

Although PPIs are effective and widely used acid-suppressing medications, there are growing concerns about their long-term use due to potential adverse effects, including increased risks of *Clostridium difficile* infection, pneumonia, and myocardial ischemia [9,10,11,12,13]. While H2RAs, alternative options, demonstrate limited efficacy, require multiple daily doses, and may lead to potential drug interactions [14,15]. Moreover, the most troubling aspect is the occurrence of tachyphylaxis within two weeks of using these drugs, which limits their role as a therapeutic agent [16].

Stillen^®^ (Stillen^®^ Dong-A ST, Seoul, Republic of Korea), extracted from *Artemisia asiatica*, demonstrates antioxidative and anti-inflammatory effects, promotes mucosal regeneration, and enhances cytoprotective prostaglandin production. Previous clinical studies demonstrated that Stillen^®^ improves endoscopic outcomes and alleviates gastrointestinal symptoms more effectively than a placebo. Its complementary effects have also been noted when used with NSAIDs [17,18]. Moreover, Stillen^®^ has been widely used as an initial gastritis mucosal protective agent without evident severe adverse events in routine clinical use [19]. However, there is limited evidence comparing Stillen^®^ with established mucosal protective agents like rebamipide. Rebamipide, along with Stillen^®^, is a widely prescribed representative mucosal protective agent in East Asian clinical practice, exerting its effects through prostaglandin synthesis, scavenging free radicals, and enhancing mucin production, thereby promoting mucosal protection and ulcer healing [20]. In addition, multiple Korean clinical trials of gastritis treatments frequently selected rebamipide and Stillen^®^ as active comparators, reflecting their well-established efficacy and recognition as standard reference drugs [21,22,23,24,25,26]. However, many Stillen^®^ trials have been conducted in Korea; not all relevant clinical trials have been disseminated through peer-reviewed journals. Relying on published evidence would risk selective availability of positive findings and create an imbalance in the evidence base compared with rebamipide, which has a larger body of published studies. To reduce publication bias and strengthen the reliability of assessment, unpublished randomized clinical trials were also included. Therefore, we conducted a systematic review and network meta-analysis of randomized clinical trials (RCTs) to determine whether Stillen^®^ is non-inferior in endoscopic improvement outcomes compared to rebamipide in patients with acute or chronic gastritis, with the goal of addressing the gap in current guidelines and informing evidence-based selection of mucosal protective therapy in clinical practice.

## 2. Materials and Methods

This study was conducted as a systematic review and network meta-analysis (NMA) to compare the treatment efficacy and safety of Stillen^®^ (60 mg, 3 times/day) versus rebamipide (100 mg, 3 times/day) in patients with gastritis. The main objective of this study was to compare efficacy. The analysis incorporated both direct and indirect evidence from RCTs. Eligible studies included RCTs evaluating the efficacy of Stillen^®^, rebamipide, or comparator interventions (e.g., placebo or other gastric mucosa-protective agents) in patients diagnosed with gastritis. The protocol was retrospectively registered on PROSPERO (CRD420251127024).

### 2.1. Outcomes

The primary outcome was the improvement rate, defined as the percentage of pa-tients with a reduction in erosion of more than 50%. Patients were classified as effective cases if they showed ≥50% reduction in initial scores at the follow-up esophagogastroduodenoscopy (EGD) two weeks after treatment initiation. Endoscopic findings were categorized as effective (4 to 1, 3 to 1, 2 to 1, or 4 to 2) and ineffective (all other outcomes). Secondary outcomes included improvement rates at 2, 3, and 4 weeks post-treatment and the cure rate of erosions, defined as the complete disappearance of all erosions, assessed by endoscopy at the same time points. For safety assessments, adverse events (AEs), serious adverse events (SAEs), adverse drug reactions (ADRs), and gastrointestinal (GI) disorders were evaluated. GI disorder was selected as a safety outcome because both Stillen^®^ and rebamipide are mucosal protective agents primarily used in the gastrointestinal tract, and their most clinically relevant adverse events are expected to occur within the GI system. As the primary objective of this study was to compare efficacy, subsequent methodological assessments of the risk of bias evaluation, certainty of evidence, and sensitivity analyses were conducted for efficacy outcomes.

### 2.2. Data Collection Methods

**Figure 1:**
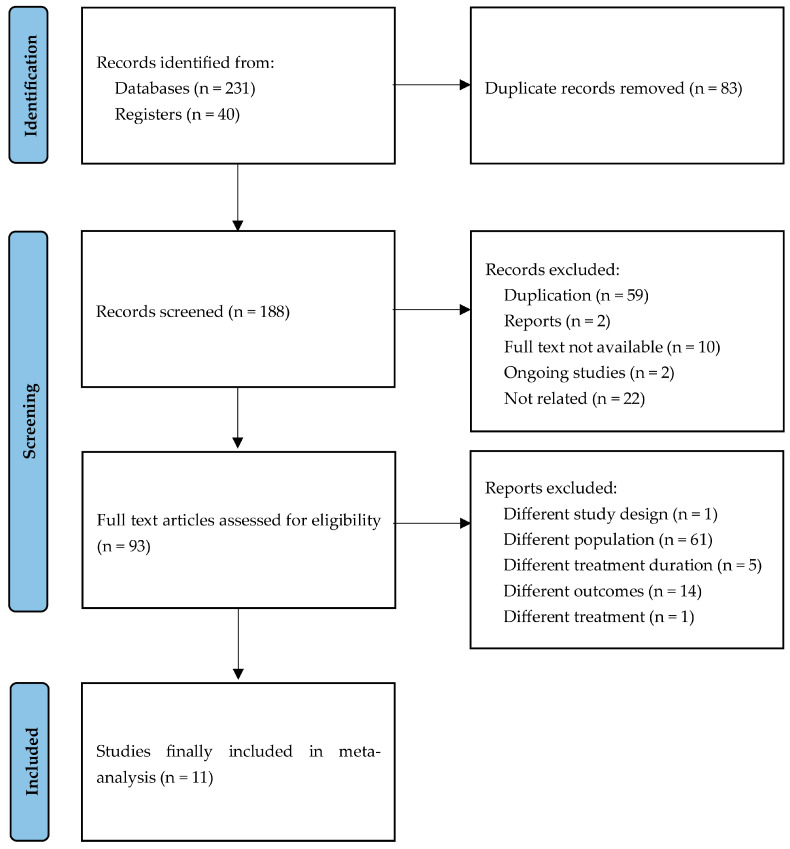
PRISMA flow diagram for identification of studies via databases and registers.

To ensure the inclusion of high-quality literature, we restricted the review to RCTs. We used “gastritis” as the keyword for the patient population and “Stillen^®^” and “rebamipide” as intervention terms. The literature was searched in PubMed, EMBASE, Cochrane Library, RISS (Research Information Sharing Service), and KoreaMed (Korean medical literature database). Additionally, trial data were searched in ClinicalTrials.gov and the International Clinical Trials Registry Platform (ICTRP) for unpublished trials. MeSH terms were used in PubMed and the Cochrane Library; Emtree terms were used in EMBASE; and keyword combinations were used in RISS. Natural language terms and Boolean operators (AND, OR, NOT) were also applied to refine the search strategy. The literature search was completed on 15 August 2025. The detailed search strategy based on the database is provided in Supplementary Table S1. The review followed the PRISMA guidelines (Supplementary Tables S2 and S3). After removing duplicates, 188 studies were screened. Among these, one unpublished RCT was identified through ICTRP. In addition, one further unpublished RCT, which was a phase II trial conducted in 1999, is being retrospectively registered on ClinicalTrials.gov. For these two studies, we contacted the pharmaceutical company (Dong-A ST, Seoul, Republic of Korea) and obtained clinical study reports. These unpublished studies were assessed with the same eligibility, extraction, and appraisal procedures as published studies. A total of 11 eligible studies, comprising both published and unpublished trials, were included in the final analysis (Figure 1).

### 2.3. Eligibility Criteria

Studies were eligible if they met the following inclusion criteria: (1) RCTs involving adult patients with acute or chronic gastritis, (2) reported improvement rate outcomes, and (3) a treatment duration of 2, 3, or 4 weeks. Studies were excluded if they (1) were not RCTs, (2) lacked assessable eligibility or outcome data, or (3) did not meet any of the inclusion criteria.

### 2.4. Study Selection

Systematically searched and retrieved titles and abstracts were independently screened by two reviewers. Full texts were retrieved for studies that met or potentially met the inclusion criteria. The same reviewers independently assessed these full texts. Disagreements were resolved by a third reviewer, but full consensus was achieved without adjudication. A flow diagram of the selection process is presented in Figure 1.

### 2.5. Data Extraction and Quality Assessment

We extracted data on study characteristics (year of publication, design, treatment duration, sample size, interventions), participant demographics (age and sex), and outcomes (primary and secondary), as well as the analysis sets used (Full Analysis Set [FAS], Per-Protocol Set [PPS]). Risk of bias was assessed using the Cochrane Risk of Bias tool [27], which evaluates the randomization process, deviations from intended interventions, missing outcome data, outcome measurement, and selective reporting. Two authors independently reviewed each domain, and there were no discrepancies between the two authors.

### 2.6. Grading of the Certainty of Evidence

We assessed the certainty of evidence (CoE) for each outcome using the Grading of Recommendations Assessment, Development, and Evaluation (GRADE) method, considering risk of bias, inconsistency, indirectness, and publication bias [28]. The level of CoE was rated as high, moderate, low, or very low. We used the free web-based version of the GRADEpro GDT, McMaster University, Hamilton, Canada; https://gradepro.org accessed on 15 August 2025.

### 2.7. Statistical Analysis

The primary analysis used a frequentist-based NMA to compare the efficacy and the safety of Stillen^®^ (60 mg, 3 times/day) and rebamipide (100 mg, 3 times/day). We evaluated the assumptions of NMA, transitivity, similarity, and consistency by examining patient demographics, treatment dosages, study design, and outcome definitions. Consistency was assessed using the decomp.design function in the netmeta package for a design-by-treatment interaction model (global inconsistency) and node-splitting method (local inconsistency) with the netsplit function in netmeta package. Heterogeneity was evaluated using the I^2^ statistic and chi-square test; values of I^2^ > 50% or *p* < 0.1 were considered significant [29]. A random-effects model was applied to account for between-study heterogeneity. Effect sizes were reported as odds ratios (ORs) with 95% two-sided confidence intervals (CIs), reflecting relative comparisons [30,31]. Based on the prior literature, the non-inferiority margin for the absolute difference in improvement rate (Stillen^®^–rebamipide) was prespecified as −0.20 to −0.14 [18,21,22,23,24,32]. Assuming a 48% improvement rate in the rebamipide group [25], this corresponds to ORs ranging from 0.42 to 0.56. For this study, we selected a conservative margin of −0.14 (OR = 0.56). A separate analysis assessed cure rates between treatments. In secondary analyses, studies were pooled separately by drug, including Stillen^®^ (90 mg, 2 times/day) and rebamipide (150 mg, 2 times/day), aligned with the dosages in the primary analysis. Pooled effect sizes and standard errors were calculated for each treatment group. Non-inferiority and difference tests were performed for improvement and cure rates, respectively. In addition to the network meta-analysis, we also presented forest plots of direct comparisons to enhance transparency and allow for assessing the consistency between direct and indirect evidence. In these direct comparisons, only the pairwise comparisons between Stillen^®^ and rebamipide were extracted in multi-arm trials that involved more than two interventions. Sensitivity analyses were conducted, restricted to studies with low RoB, to published studies, and to double-blind trials. For missing data, we extracted outcome results based on both the FAS and the PPS, as reported in each trial. No additional statistical imputation was performed at the review level, and analyses were conducted using the available data. For multi-arm trials, each comparison was included in the network meta-analysis while accounting for the correlation between effect estimates from the same trial. In the frequentist framework, this was handled using the netmeta package in R, which implements variance adjustment for multi-arm trials. The statistical analysis was conducted in accordance with the guidelines of the Health Insurance Review and Assessment Service of Republic of Korea [33,34], and all analyses were conducted for FAS and PPS using R (version 4.4.2) with the netmeta (version 2.9.0) and meta (version 8.0.2) packages. The analytic codes used for this study are available in the “NMA-of-Stillen-vs-Rebamipide” repository: https://github.com/snowball271/NMA-of-Stillen-vs-Rebamipide- (accessed on 15 August 2025).

## 3. Results

### 3.1. Study Selection and Characteristics

A total of 188 studies were identified after removing duplicates. Following screening, 177 studies were excluded. Of the 93 full-text articles assessed for eligibility, the most common reason for exclusion was different populations (*n* = 61). Finally, 11 eligible RCTs were included in the final analysis (Figure 1). All studies were randomized, multi-center trials; two were single-blinded and nine were double-blinded. Regarding the clinical trial phase, three were phase 2, four were phase 3, three were phase 4, and one was of an unknown phase. Treatment durations were 2 weeks (*n* = 7), 3 weeks (*n* = 1), and 4 weeks (*n* = 3). Study sizes varied across trials, and two studies did not report the cure rate at 2 weeks (Table 1).

All studies enrolled patients with acute or chronic gastritis who were endoscopy-confirmed for one or more erosions. Major exclusion criteria included a history of peptic ulcer or reflux esophagitis, as well as the use of medications or the presence of comorbid conditions that could affect study outcomes. Detailed inclusion and exclusion criteria are summarized in Supplementary Table S4.

Of the 11 RCTs, 9 were included in the primary analysis, excluding 2 comparative studies involving drugs with the same active ingredient. Five studies had a treatment duration of 2 weeks, and among these, three that included Stillen^®^ or rebamipide were incorporated into the two-week outcome analysis. All nine were included in the pooled 2, 3, and 4 weeks primary analysis for efficacy outcomes. All 11 studies were included in the secondary analysis. In the safety outcomes, two studies could not be connected within the networks, and nine studies were included in the safety evaluation.

### 3.2. Efficacy Outcomes

#### 3.2.1. Primary Analysis

##### Improvement Rate

**Figure 2:**
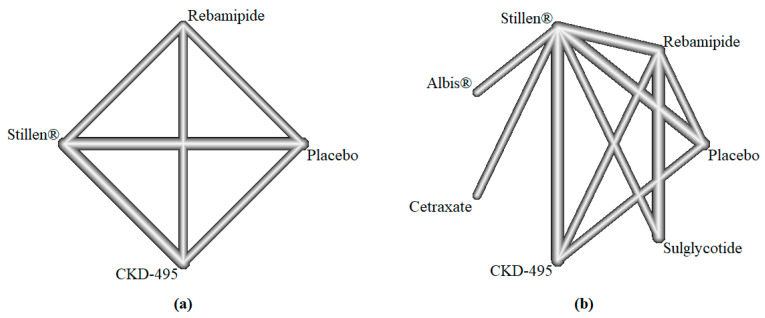
Network plots of included studies for the improvement rate. (**a**) Treatment duration of 2 weeks. (**b**) Treatment duration of 2, 3, and 4 weeks. CKD-495: a component extracted from *Cinnamomum cassia*.

Network plots are presented in Figure 2. At 2 weeks (*n* = 3), the OR (95% CI) for improvement with Stillen^®^ versus rebamipide was 1.11 (0.88, 1.39) in FAS and 1.09 (0.85, 1.41) in PPS, indicating non-inferiority of Stillen^®^. When pooled across 2, 3, and 4 weeks (*n* = 9), the ORs were 0.99 (0.88, 1.10) in FAS and 0.98 (0.87, 1.11) in PPS. All analyses supported the non-inferiority of Stillen^®^ compared to rebamipide in terms of improvement rate (Table 2). Across the improvement rates, the ORs ranged from 0.98 to 1.11, with a lower bound of the 95% CIs between 0.85 and 0.88, further reinforcing the robustness of non-inferiority for the improvement rate. Forest plots of the direct comparisons for the improvement rate are shown in supplementary Figure S1, and the network meta-analysis combining both direct and indirect evidence yielded effect estimates that were consistent with the direct comparisons regarding OR and the lower bound of the 95% CI for OR.

##### Cure Rate

At 2 weeks (*n* = 1), the OR (95% CI) for cure rate with Stillen^®^ versus rebamipide was 1.03 (0.84, 1.26) in FAS and 1.00 (0.81, 1.24) in PPS, with no significant difference between treatments. When pooled across 2, 3, and 4 weeks (*n* = 7), the ORs were 0.96 (0.88, 1.05) in FAS and 0.96 (0.87, 1.06) in PPS, again indicating no significant difference (Table 2). The network meta-analysis also provided the estimate of cure rates that were largely consistent with the direct comparisons regarding OR and the 95% CI for OR (Supplementary Figure S1).

#### 3.2.2. Secondary Analysis

##### Improvement Rate

At 2 weeks (*n* = 7), pooled improvement rates were 0.46 (Stillen^®^) and 0.42 (rebamipide) in FAS (difference = 0.04; 95% CI: (−0.05, 0.12)) and 0.47 (Stillen^®^) and 0.42 (rebamipide) in PPS (difference = 0.05; 95% CI: (−0.04, 0.15)). Non-inferiority was demonstrated in both analysis sets. When pooled across all durations (2, 3, and 4 weeks) (*n* = 11), the improvement rates were 0.45 (Stillen^®^) and 0.49 (rebamipide) in FAS (difference = −0.04; 95% CI: (−0.139, 0.05)) and 0.47 (Stillen^®^) and 0.50 (rebamipide) in PPS (difference = −0.04; 95% CI: (−0.138, 0.07)), supporting non-inferiority (Table 3).

##### Cure Rate

At 2 weeks (*n* = 5), the pooled cure rates were 0.39 (Stillen^®^) and 0.36 (rebamipide) in FAS (difference = 0.04; 95% CI: (−0.05, 0.13)), and 0.41 (Stillen^®^) and 0.35 (rebamipide) in PPS (difference = 0.05; 95% CI: (−0.04, 0.15)). The differences were not statistically significant in either set. When pooled across 2, 3, and 4 weeks (*n* = 9), the cure rates were 0.38 (Stillen^®^) and 0.40 (rebamipide) in FAS (difference = −0.02; 95% CI: (−0.14, 0.11)), and 0.40 (Stillen^®^) and 0.40 (rebamipide) in PPS (difference = 0.01; 95% CI: (−0.11, 0.12)), again showing no significant difference (Table 3).

### 3.3. Safety Outcomes

Safety outcomes were evaluated based on the safety set from each study. The incidence of AEs (8 patients at 2, 3, and 4 weeks), ADRs (2 patients at 2 weeks; 6 patients at 2, 3 and 4 weeks), GI AEs (6 patients at 2, 3, and 4 weeks), and GI ADRs (2 patients at 2 weeks; 5 patients at 2, 3, and 4 weeks) was low and did not differ significantly between Stillen^®^ and rebamipide (Table 4). SAEs occurred in one patient treated with Stillen^®^ treatment and two patients treated with rebamipide. These network-meta-analysis-based results were consistent with the direct comparisons (Supplementary Figure S2).

At 2 weeks, AEs and GI disorder AEs were reported in two and one trials, respectively, and network meta-analysis between Stillen^®^ and rebamipide was impossible due to the absence of a connected network.

### 3.4. Sensitivity Analysis

Sensitivity analyses of improvement rate and cure rate are shown in Supplementary Table S5. For the improvement rate, three double-blind and two low-risk-of-bias trials, as well as two published studies, reported outcomes at 2 weeks. In addition, seven double-blind trials, six low-risk-of-bias trials, and seven published studies reported improvement rates at 2, 3, and 4 weeks. For the cure rate, one double-blind trial, one low-risk-of-bias trial, and one published study reported outcomes at 2 weeks, while five double-blind trials, five low-risk-of-bias trials, and six published studies reported outcomes at 2, 3, and 4 weeks. Overall, the results of three sensitivity analyses restricted to double-blind trials, studies with low risk of bias, and published studies were consistent with those obtained when including single-blind trials, studies with some concerns for risk of bias, and unpublished studies.

### 3.5. Assumptions of NMA and Study Quality

**Figure 3:**
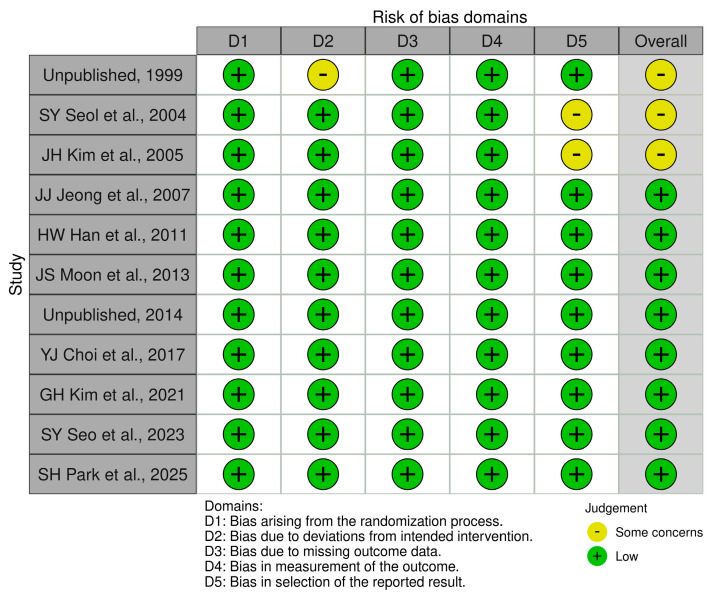
Risk of bias assessment [17,18,21,22,23,24,25,26,32,35,36] (Cochran Risk of Bias Tool [27]).

Comparable baseline characteristics, study designs, and inclusion/exclusion criteria across the included RCTs (Table 1, Supplementary Table S4) supported the assumptions of transitivity and similarity. Consistency was confirmed using both global and local statistical methods (all *p* > 0.05). In the assessment of risk of bias, all 11 studies were judged to be at low risk for bias arising from the randomization process, bias due to missing outcome data, and bias in outcome measurement. One study showed some potential bias due to deviations from the intended intervention, as did two studies in their selection of the reported results. Overall, eight studies were rated as having low risk of bias and three studies posed some concerns, indicating that the overall risk of bias across the included studies was low (Figure 3).

### 3.6. GRADE Assessment

Using the GRADE approach, the certainty of evidence was judged as high for improvement rate at 2 weeks and at 2, 3, and 4 weeks of treatment and for cure rate at 2, 3, 4 weeks of treatment but moderate for cure rate at 2 weeks (Supplementary Table S6). Improvement rates derived from Stillen^®^ compared to rebamipide satisfied the non-inferiority, and we did not downgrade for risk of bias, inconsistency, indirectness, and imprecision. However, the cure rate at 2 weeks of treatment was derived from a single small RCT with wide confidence intervals, therefore, we downgraded for imprecision. Publication bias in other considerations was not formally assessed due to the limited number of studies, although no apparent publication bias was observed (Supplementary Table S6).

## 4. Discussion

This systematic review and network meta-analysis demonstrated that Stillen^®^ was non-inferior in improvement rates to the standard mucosal protective agent rebamipide across multiple treatment durations (2, 3, and 4 weeks) in patients with acute or chronic gastritis. Notably, the primary outcome of a 2-week improvement rate met the predefined non-inferiority margin, further confirming that Stillen^®^ provides a comparable efficacy profile to the established treatment. The pooled analysis across different durations highlights the antioxidative and anti-inflammatory mechanisms by which Stillen^®^ exerts its effects. Additionally, the comparison of cure rates revealed no significant difference between Stillen^®^ and rebamipide, suggesting that both agents promote mucosal healing with similar efficacy. These findings align with earlier small-scale RCTs and provide a more robust, integrated perspective on the performance of Stillen^®^ across diverse patient populations. We further synthesized direct and indirect comparisons, and the direct comparison results consistently aligned with the network meta-analysis, indicating no substantial alteration of conclusions by indirect evidence.

Multiple sensitivity analyses, excluding single-blind trials, studies not judged at low risk of bias, and unpublished data, yielded consistent results with the primary analysis. According to the GRADE assessment, although the cure rate at 2 weeks was rated as moderate due to imprecision, warranting cautious interpretation, the certainty of evidence was rated as high for most improvement and cure rate outcomes, supporting the robustness of our conclusions while warranting cautious interpretation.

While a study directly comparing Stillen^®^ and rebamipide at 4 weeks was conducted, it lacked sufficient statistical power to confirm non-inferiority [24]. Therefore, our systematic review employed an indirect comparison through a comprehensive literature search to assess the non-inferiority of Stillen^®^ in terms of improvement rates at 2 weeks. This approach supports the conclusion that Stillen^®^ provides efficacy comparable to rebamipide, particularly in the early treatment period. These findings are consistent with previous evidence suggesting that Stillen^®^, like other mucosal protectants such as rebamipide, enhances protective factors in the gastric environment through multiple mechanisms, including suppression of neutrophil activity, antioxidant effects, inhibition of NF-κB nuclear translocation, and early induction of heat shock proteins that contribute to cellular defense [17,18,22,37,38]. Stillen^®^ is also approved for prophylactic use against NSAID-induced gastritis, reflecting its role in mitigating prostaglandin depletion caused by prolonged NSAID administration [19,37]. Given the multifactorial nature of chronic gastritis, combining Stillen^®^ with PPIs or *Helicobacter pylori* eradication regimens could further enhance mucosal healing.

To provide a comprehensive evaluation of relative efficacy, our analysis synthesized both direct and indirect comparisons. The results of the direct comparisons were consistent with those from the network meta-analysis, indicating that the inclusion of indirect evidence did not substantially alter the conclusions. This consistency, along with the findings from sensitivity analyses, strengthens the validity of our findings and supports the robustness of the network meta-analysis approach. Comprehensive sensitivity analyses were conducted to address potential concerns regarding robustness. The exclusion of single-blind trials, studies with a non-low risk of bias, and unpublished data did not induce any substantial changes in the overall results. Although this analysis integrated both direct and indirect comparisons using a network meta-analytic approach, the number of studies contributing to each time point was limited. The heterogeneity across studies, such as in terms of study size, improvement rates, and cure rates, may complicate the interpretation of the overall findings. In addition, our analysis did not incorporate patient-level data (IPD) and did not have sufficient available number of studies. Hence, we could not perform deeper subgroup or meta-regression analyses to explore effect modifiers (e.g., age, baseline erosion severity). Furthermore, although there were differences in the definitions of the non-inferiority margin across studies, this study assessed non-inferiority using the most conservative margin.

## 5. Conclusions

Stillen^®^ demonstrated non-inferiority to rebamipide in terms of endoscopic improvement of erosions in patients with acute or chronic gastritis. The cure rates and safety outcomes were comparable between the two agents. These results suggest that Stillen^®^ may be considered a clinically viable alternative to rebamipide in the treatment of erosive gastritis as the mucosal protective agent.

## Figures and Tables

**Table 1:** Characteristics of included studies.

Study ID	Study Arms	Study Design	Phase	*n*	Sex (Male), (%)	Age (Years), Mean ± SD	Treatment Duration
Unpublished, 1999 [35]	Placebo, Stillen^®^ 60 mg, 3 times/day, Stillen^®^ 120 mg, 3 times/day	Double-blind, Randomized, Multi-center trial	II	176	101 (57.4)	45.9 ± 11.30	2 weeks
SY Seol et al., 2004 [17]	Stillen^®^ 60 mg, 3 times/day, Stillen^®^ 120 mg, 3 times/day, Cetraxate 200 mg, 3 times/day	Double-blind, Randomized, Multi-center trial	III	512	253 (49.4)	45.7 ± 11.56	2 weeks
HW Han et al., 2011 [21]	Albis^® a^ 1 tab, 2 times/day, Stillen^®^ 60 mg 3, times/day	Double-blind, Randomized, Multi-center trial	II	229	118 (51.5)	44.6 ± 10.20	2 weeks
YJ Choi et al., 2017 [18]	Stillen^®^ 60 mg, 3 times/day, Stillen^®^ 90 mg, 2 times/day	Double-blind, Randomized, Multi-center trial	III	434	167 (38.5)	45.7 ± 13.05	2 weeks
GH Kim et al., 2021 [22]	Rebamipide 150 mg, 2 times/day, Rebamipide 100 mg, 3 times/day	Double-blind, Randomized, Multi-center trial	III	454	187 (41.2)	47.0 ± 12.20	2 weeks
SY Seo et al., 2023 [26]	CKD-495 75 mg, 3 times/day, Stillen^®^ 60 mg, 3 times/day	Double-blind, Randomized, Multi-center trial	III	220	85 (38.6)	48.1 ± 13.20	2 weeks
SH Park et al., 2025 [25]	Placebo, CKD-495 75 mg, 3 times/day, CKD-495 150 mg, 3 times/day, Stillen^®^ 60 mg, 3 times/day, Rebamipide 100 mg, 3 times/day	Double-blind, Randomized, Multi-center trial	II	233	122 (52.7)	48.6 ± 16.40	2 weeks
JJ Jeong et al., 2007 [23]	Sulglycotide 200 mg, 3 times/day, Rebamipide 100 mg, 3 times/day	Single-blind, Randomized, Multi-center trial	IV	73	23 (31.5)	49.7 ± 10.00	3 weeks
JH Kim et al., 2005 [36]	Stillen^®^ 60 mg, 3 times/day, Sulglycotide 200 mg, 3 times/day	Single-blind, Randomized, Multi-center trial	IV	150	66 (44.0)	52.7 ± 11.40	4 weeks
JS Moon et al., 2013 [24]	Sulglycotide 200 mg, 3 times/day, Rebamipide 100 mg, 3 times/day	Double-blind, Randomized, Multi-center trial	UK	197	66 (33.5)	49.9 ± 12.40	4 weeks
Unpublished, 2014 [32]	Stillen^®^ 60 mg, 3 times/day, Rebamipide 100 mg, 3 times/day	Double-blind, Randomized, Multi-center trial	IV	266	103 (38.7)	42.4 ± 12.19	4 weeks
**Study ID**	**Study Arms**	**N1, N2**	**Improvement** **n1 (%), n2 (%)**			**Cure** **n1 (%), n2 (%)**	
Unpublished, 1999 [35]	Placebo, Stillen^®^ 60 mg, 3 times/day, Stillen^®^ 120 mg, 3 times/day	50, 42 52, 44 49, 39	8 (16.00), 6 (14.30) 25 (48.10), 22 (50.00) 17 (34.70), 14 (35.90)			N/A, N/A N/A, N/A N/A, N/A	
SY Seol et al., 2004 [17]	Stillen^®^ 60 mg, 3 times/day, Stillen^®^ 120 mg, 3 times/day, Cetrarate 200 mg, 3 times/day	186, 171 140, 120 186, 166	118 (63.40), 115 (67.30) 81 (57.90), 78 (65.00) 83 (44.60), 77 (46.40)			97 (52.20), 95 (55.60) 72 (51.40), 69 (57.50) 65 (35.00), 59 (35.50)	
HW Han et al., 2011 [21]	Albis^®^ 1 tab, 2 times/day, Stillen^®^ 60 mg, 3 times/day	116, 87 113, 96	41 (35.30), 37 (42.50) 39 (34.50), 38 (39.60)			32 (27.60), 28 (32.20) 31 (27.40), 30 (31.30)	
YJ Choi et al., 2017 [18]	Stillen^®^ 60 mg, 3 times/day, Stillen^®^ 90 mg, 2 times/day	212, 199 209, 197	90 (42.50), 86 (43.20) 88 (42.10), 82 (41.60)			79 (37.30), 75 (37.70) 78 (37.30), 73 (37.10)	
GH Kim et al., 2021 [22]	Rebamipide 150 mg, 2 times/day, Rebamipide 100 mg, 3 times/day	229, 224 224, 216	91 (39.70), 88 (39.30) 98 (43.80), 94 (43.70)			79 (34.50), 76 (33.90) 80 (35.70), 77 (35.80)	
SY Seo et al., 2023 [26]	CKD-495 75 mg, 3 times/day, Stillen^®^ 60 mg, 3 times/day	111, 109 109, 102	62 (55.86), 60 (54.63) 43 (39.45), 39 (38.24)			N/A, N/A N/A, N/A	
SH Park et al., 2025 [25]	Placebo, CKD-495 75 mg, 3 times/day, CKD-495 150 mg, 3 times/day, Stillen^®^ 60 mg, 3 times/day, Rebamipide 100 mg, 3 times/day	47, 45 52, 48 44, 41 48, 43 42, 38	21 (45.00), 20 (44.44) 38 (73.00), 36 (75.00) 18 (41.00), 15 (36.59) 25 (52.00), 22 (51.16) 20 (48.00), 19 (50.00)			20 (43.00), 19 (42.00) 36 (69.00), 34 (71.00) 18 (41.00), 15 (37.00) 21 (44.00), 18 (42.00) 17 (41.00), 16 (42.00)	
JJ Jeong et al., 2007 [23]	Sulglycotide 200 mg, 3 times/day, Rebamipide 100 mg, 3 times/day	36, 27 37, 32	18 (50.00), 17 (63.00) 20 (54.10), 20 (62.50)			8 (22.20), 8 (29.60) 8 (21.60), 7 (21.60)	
JH Kim et al., 2005 [36]	Stillen^®^ 60 mg, 3 times/day, Sulglycotide 200 mg, 3 times/day	74, 56 76, 59	24 (32.40), 22 (39.30) 29 (38.20), 28 (47.50)			15 (20.30), 15 (26.80) 19 (25.00), 19 (32.20)	
JS Moon et al., 2013 [24]	Sulglycotide 200 mg, 3 times/day, Rebamipide 100 mg, 3 times/day	98, 88 99, 90	51 (52.00), 47 (53.40) 60 (60.60), 55 (61.61)			43 (43.90), 40 (45.50) 53 (53.50), 48 (53.30)	
Unpublished, 2014 [32]	Stillen^®^ 60 mg, 3 times/day, Rebamipide 100 mg, 3 times/day	136, 115 126, 104	70 (51.50), 56 (48.70) 69 (54.80), 55 (52.90)			67 (49.30), 57 (47.00) 66 (52.40), 52 (50.00)	
**Study ID**	**Study Arms**	***n***	**AE** ***n* (%)**		**ADR** ***n* (%)**	**GI disorder AE** ***n* (%)**	**GI disorder ADR** ***n* (%)**
Unpublished, 1999 [35]	Placebo, Stillen^®^ 60 mg, 3 times/day, Stillen^®^ 120 mg, 3 times/day	60 60 56	4 (6.67) 1 (1.67) 0 (0.00)		3 (5.00) 1 (1.67) 0 (0.00)	4 (6.67) 1 (1.67) 0 (0.00)	3 (5.00) 1 (1.67) 0 (0.00)
SY Seol et al., 2004 [17]	Stillen^®^ 60 mg, 3 times/day, Stillen^®^ 120 mg, 3 times/day, Cetraxate 200 mg, 3 times/day	186 140 186	9 (4.84) 0 (0.00) 6 (3.23)		9 (4.84) 0 (0.00) 5 (2.69)	6 (3.23) 0 (0.00) 2 (1.08)	6 (3.23) 0 (0.00) 2 (1.08)
HW Han et al., 2011 [21]	Albis^®^ 1 tab, 2 times/day, Stillen^®^ 60 mg, 3 times/day	115 116	9 (7.83) 10 (8.62)		1 (0.87) 4 (3.45)	N/A N/A	N/A N/A
YJ Choi et al., 2017 [18]	Stillen^®^ 60 mg, 3 times/day, Stillen^®^ 90 mg, 2 times/day	217 215	19 (8.76) 18 (8.37)		4 (1.84) 5 (2.33)	11 (5.07) 11 (5.12)	2 (0.92) 5 (2.33)
GH Kim et al., 2021 [22]	Rebamipide 150 mg, 2 times/day, Rebamipide 100 mg, 3 times/day	236 234	24 (10.17) 20 (8.55)		17 (7.20) 12 (5.13)	N/A N/A	4 (1.69) 3 (1.28)
SY Seo et al., 2023 [26]	CKD-495 75 mg, 3 times/day, Stillen^®^ 60 mg, 3 times/day	122 120	4 (3.28) 5 (4.17)		N/A N/A	N/A N/A	N/A N/A
SH Park et al., 2025 [25]	Placebo, CKD-495 75 mg, 3 times/day, CKD-495 150 mg, 3 times/day, Stillen^®^ 60 mg, 3 times/day, Rebamipide 100 mg, 3 times/day	48 54 58 48 45	N/A N/A N/A N/A N/A		2 (4.17) 3 (5.56) 4 (6.90) 3 (6.25) 4 (8.89)	N/A N/A N/A N/A N/A	1 (2.08) 3 (5.56) 2 (3.45) 2 (4.17) 4 (8.89)
JJ Jeong et al., 2007 [23]	Sulglycotide 200 mg, 3 times/day, Rebamipide 100 mg, 3 times/day	36 37	2 (5.56) 4 (10.81)		N/A N/A	2 (5.56) 4 (10.81)	N/A N/A
JH Kim et al., 2005 [36]	Stillen^®^ 60 mg, 3 times/day, Sulglycotide 200 mg, 3 times/day	74 76	8 (10.81) 15 (19.74)		N/A N/A	8 (10.81) 10 (13.16)	N/A N/A
JS Moon et al., 2013 [24]	Sulglycotide 200 mg, 3 times/day, Rebamipide 100 mg, 3 times/day	100 102	11 (11.00) 12 (11.76)		2 (2.00) 2 (1.96)	4 (4.00) 2 (1.96)	2 (2.00) 2 (1.96)
Unpublished, 2014 [32]	Stillen^®^ 60 mg, 3 times/day, Rebamipide 100 mg, 3 times/day	137 129	18 (13.14) 19 (14.73)		2 (1.46) 3 (2.33)	13 (9.49) 9 (6.98)	1 (0.73) 2 (1.55)

*n* and descriptive statistics of sex and age are based on baseline characteristics presented in each study. SD: Standard deviation. UK: Unknown. CKD-495: A component extracted from *Cinnamomum cassia.* N1: Number of patients in Full Analysis Set (FAS), N2: Number of patients in Per-Protocol Set (PPS), n1: Number of improved patients in FAS, n2: Number of improved patients in PPS. N/A: Not applicable due to no data. N: Number of patients in safety set, *n*: Number of patients who occurred. AE: Adverse event, ADR: Adverse drug reaction, GI: Gastrointestinal. ^a^: Daewoong pharmaceutical, Seoul, Republic of Korea.

**Table 2:** Primary analysis: comparison of the improvement rate and the cure rate between Stillen^®^ and rebamipide.

Outcome	Treatment Duration	Analysis Set	OR	95% CI for OR
Improvement rate	2 weeks	FAS	1.11	(0.88, 1.39) ^S_NE)^
Improvement rate	2 weeks	PPS	1.09	(0.85, 1.41) ^S_NE)^
Improvement rate	2, 3, and 4 weeks	FAS	0.99	(0.88, 1.10) ^S_NE)^
Improvement rate	2, 3, and 4 weeks	PPS	0.98	(0.87, 1.11) ^S_NE)^
Cure rate	2 weeks	FAS	1.03	(0.84, 1.26) ^NS_D)^
Cure rate	2 weeks	PPS	1.00	(0.81, 1.24) ^NS_D)^
Cure rate	2, 3, and 4 weeks	FAS	0.96	(0.88, 1.05) ^NS_D)^
Cure rate	2, 3, and 4 weeks	PPS	0.96	(0.87, 1.06) ^NS_D)^

OR: Odds ratio of Stillen^®^ compared to rebamipide, CI: Confidence interval. FAS: Full analysis set, PPS: Per-protocol set. ^S_NE)^ Significance for non-inferiority of Stillen^®^ compared to rebamipide at one-sided 2.5% significance level (non-inferiority margin = 0.56). ^NS_D)^ No significance for the difference between Stillen^®^ and rebamipide at a two-sided 5% significance level

**Table 3:** Secondary analysis: Comparison of the improvement rate and the cure rate between Stillen^®^ and rebamipide.

Outcome	Treatment Duration	Analysis Set	Stillen^®^	Rebamipide	RD	95% CI for RD
Improvement rate	2 weeks	FAS	0.46	0.42	0.04	(–0.05, 0.12) ^S_NE)^
Improvement rate	2 weeks	PPS	0.47	0.42	0.05	(–0.04, 0.15) ^S_NE)^
Improvement rate	2, 3, and 4 weeks	FAS	0.45	0.49	–0.04	(–0.139, 0.05) ^S_NE)^
Improvement rate	2, 3, and 4 weeks	PPS	0.47	0.50	–0.04 ^1)^	(–0.138, 0.07) ^S_NE)^
Cure rate	2 weeks	FAS	0.39	0.36	0.04 ^2)^	(–0.05, 0.13) ^NS_D)^
Cure rate	2 weeks	PPS	0.41	0.35	0.05 ^3)^	(–0.04, 0.15) ^NS_D)^
Cure rate	2, 3, and 4 weeks	FAS	0.38	0.40	–0.02	(–0.14, 0.11) ^NS_D)^
Cure rate	2, 3, and 4 weeks	PPS	0.40	0.40	0.01 ^4)^	(–0.11, 0.12) ^NS_D)^

RD: Risk difference (Stillen^®^–rebamipide), CI: Confidence interval. FAS: Full analysis set, PPS: Per-protocol set. ^S_NE)^ Significance for non-inferiority of Stillen^®^ compared to rebamipide at one-sided 2.5% significance level (non-inferiority margin –0.14). ^NS_D)^ No significance for the difference between Stillen^®^ and rebamipide at a two-sided 5% significance level. ^1)^ The rounded value of –0.036, ^2)^ the rounded value of 0.039, ^3)^ the rounded value of 0.054, ^4)^ the rounded value of 0.005.

**Table 4:** Safety outcomes.

Outcome	Treatment Duration	OR	95% CI for OR
AE	2, 3, and 4 weeks	0.96	(0.89, 1.03) ^NS_D)^
ADR	2 weeks	0.95	(0.86, 1.05) ^NS_D)^
ADR	2, 3, and 4 weeks	0.99	(0.96, 1.02) ^NS_D)^
GI disorder AE	2, 3, and 4 weeks	1.02	(0.96, 1.08) ^NS_D)^
GI disorder ADR	2 weeks	0.94	(0.85, 1.04) ^NS_D)^
GI disorder ADR	2, 3, and 4 weeks	0.98	(0.94, 1.02) ^NS_D)^

OR: Odds ratio of Stillen^®^ compared to rebamipide, CI: Confidence interval. AE: Adverse event, ADR: Adverse drug reaction, GI: Gastrointestinal. ^NS_D)^ No significance for the difference between Stillen^®^ and rebamipide at a two-sided 5% significance level.

## Data Availability

The authors confirm that the data supporting the findings of this study are available within the article.

## Supplementary Materials

The following supporting information can be downloaded at: https://www.mdpi.com/article/10.3390/jcm14176209/s1, Table S1: Database search strategies. Table S2: PRISMA 2020 Checklist. Table S3: PRISMA 2020 abstract checklist. Table S4: Inclusion and exclusion criteria of included studies. Table S5: Sensitivity analysis. Table S6: Grade assessment. Figure S1: Forest plot: Direct comparisons in efficacy outcomes. Figure S2: Forst plot: Direct comparisons in safety outcomes. Figure S3: Funnel plot.

## Abbreviations

The following abbreviations are used in this manuscript:

RCT	Randomized controlled trial
H2RAs	Histamine H2 Receptor Antagonists
NSAIDs	Nonsteroidal anti-inflammatory drugs
PPIs	Proton pump inhibitors
OR	Odds ratio
CI	Confidence interval
FAS	Full analysis set
PPS	Per-protocol set
ACG	American College of Gastroenterology
APAGE	Asian Pacific Association of Gastroenterology
GERD	Gastroesophageal reflux disease
NMA	Network Meta-Analysis
EGD	Esophagogastroduodenoscopy
AE	Adverse event
SAE	Serious adverse event
ADR	Adverse drug reaction
GI	Gastrointestinal
RISS	Research Information Sharing Service
KoreaMed	Korean medical literature database
ICTRP	International Clinical Trials Registry Platform
CoE	Certainty of evidence
GRADE	Grading of Recommendations Assessment, Development, and Evaluation
CKD-495	Component extracted from *Cinnamomum cassia*
RD	Risk difference
IPD	Individual patient data
