# Supplementary material for: Comparative Efficacy and Safety of Stillen® and Rebamipide in Patients with Acute or Chronic Gastritis: A Systematic Review and Network Meta-Analysis of Randomized Controlled Trials
Source: J Clin Med. 2025 Sep 2;14(17):6209. doi: 10.3390/jcm14176209 (PMC12429379; doi:10.3390/jcm14176209)
Supplement: Supplementary file 1 [file jcm-14-06209-s001.zip › jcm-3754388-supplementary.pdf]

Supplementary Table S1. Database search strategies.

Stillen®

Database	Full Search Strategy
PubMed	#1 "Gastritis"[Mesh]<br>#2 "Gastritis"[TW] OR "Gastritides"[TW] OR ... OR "Acute gastritis"[TW]<br>#3 "erosive gastritis"[TW] OR "gastritis, erosive"[TW]<br>#4 "chronic gastritis"[TW] OR "gastritis, chronic"[TW] ...<br>#5 "Gastritis, Atrophic"[Mesh]<br>#6 "Gastritis, Atrophic"[TW] OR "autoimmune gastritis"[TW] ...<br>#7 #1 OR #2 OR #3 OR #4 OR #5 OR #6<br>#8 "DA 9601" [Supplementary Concept]<br>#9 "DA 9601"[TW] OR "DA-9601"[TW] OR "Stillen"<br>#10 "DA-5204" [Supplementary Concept] OR "DA-5204"[TW] OR "Stillen 2X"<br>#11 "eupatilin" [Supplementary Concept]<br>#12 "eupatilin"[TW] OR "5,7-dihydroxy-3',4',6-trimethoxyflavone"[TW]<br>#13 "jaceosidin" [Supplementary Concept] OR "jaceosidin"[TW]<br>#14 "Artemisia"[Mesh]<br>#15 "Artemisia"[TW] OR "Mugwort"[TW] OR "Wormwood"[TW] OR ... OR "Artemisia princeps"[TW]<br>#16 #8 OR #9 OR #10 OR #11 OR #12 OR #13 OR #14 OR #15<br>#17 #7 AND #16<br>#18 #17 AND (randomized controlled trial filter)
EMBASE	#1 "gastritis"/exp OR "erosive gastritis"/exp OR "chronic gastritis"/exp<br>#2 "Gastritis":ti,ab,kw,de OR "Gastritides":ti,ab,kw,de OR ... OR "Acute gastritis":ti,ab,kw,de<br>#3 "erosive gastritis":ti,ab,kw,de OR "gastritis, erosive":ti,ab,kw,de<br>#4 "chronic gastritis":ti,ab,kw,de OR "gastritis, chronic":ti,ab,kw,de<br>#5 "atrophic gastritis"/exp<br>#6 "Gastritis, Atrophic":ti,ab,kw,de OR "autoimmune gastritis":ti,ab,kw,de OR ...<br>#7 #1 OR #2 OR #3 OR #4 OR #5 OR #6<br>#8 "DA 9601":ti,ab,kw,de OR "DA-9601":ti,ab,kw,de OR "Stillen"<br>#9 "DA-5204":ti,ab,kw,de OR "Stillen 2X"<br>#10 "eupatilin":ti,ab,kw,de<br>#11 "jaceosidin":ti,ab,kw,de<br>#12 "Artemisia"/exp<br>#13 "Artemisia":ti,ab,kw,de OR "Mugwort":ti,ab,kw,de OR "Wormwood":ti,ab,kw,de OR ...<br>#14 #8 OR #9 OR #10 OR #11 OR #12 OR #13<br>#15 #7 AND #14<br>#16 #15 AND [randomized controlled trial]/lim
Cochrane Library	#1 [mh "Gastritis"]<br>#2 "Gastritis":ti,ab,kw OR "gastric inflammation":ti,ab,kw OR ... OR "Acute gastritis":ti,ab,kw<br>#3 "erosive gastritis":ti,ab,kw OR "gastritis, erosive":ti,ab,kw<br>#4 "chronic gastritis":ti,ab,kw OR "gastritis, chronic":ti,ab,kw<br>#5 [mh "Gastritis, Atrophic"]<br>#6 "Gastritis, Atrophic":ti,ab,kw OR "autoimmune gastritis":ti,ab,kw OR ...<br>#7 #1 OR #2 OR #3 OR #4 OR #5 OR #6<br>#8 "DA 9601":ti,ab,kw OR "DA-9601":ti,ab,kw OR "Stillen"<br>#9 "DA-5204":ti,ab,kw OR "Stillen 2X"

	#10 "eupatilin":ti,ab,kw OR "5,7-dihydroxy-3',4',6-trimethoxyflavone":ti,ab,kw<br>#11 "jaceosidin":ti,ab,kw<br>#12 [mh "Artemisia"]<br>#13 "Artemisia":ti,ab,kw OR "Mugwort":ti,ab,kw OR "Wormwood":ti,ab,kw OR ...<br>#14 #8 OR #9 OR #10 OR #11 OR #12 OR #13<br>#15 #7 AND #14<br>#16 #15 / Trials
ClinicalTrials.gov	"Gastritis" OR "Gastritis, Atrophic" AND ("DA-9601" OR "DA-5204" OR "Stillen" OR "eupatilin" OR "jaceosidin" OR "Artemisia")
ICTRP	(Gastritis OR "Atrophic Gastritis") AND ("DA-9601" OR "DA-5204" OR "Stillen" OR "eupatilin" OR "jaceosidin" OR "Artemisia")
RISS	(Gastritis OR 위염) AND (DA-9601 OR DA-5204 OR Stillen OR eupatilin OR jaceosidin OR Artemisia)
KoreaMed	(Gastritis[TIAB] OR 위염[TIAB]) AND (DA-9601[TIAB] OR DA-5204[TIAB] OR Stillen[TIAB] OR eupatilin[TIAB] OR jaceosidin[TIAB] OR Artemisia[TIAB])

Rebamipide

**Database**	**Full Search Strategy**
PubMed	#1 "Gastritis"[Mesh]<br>#2 "Gastritis":ti,ab,kw,de OR "Gastritides":ti,ab,kw,de OR ... OR "Acute gastritis":ti,ab,kw,de<br>#3 "erosive gastritis":ti,ab,kw,de OR "gastritis, erosive":ti,ab,kw,de<br>#4 "chronic gastritis":ti,ab,kw,de OR "gastritis, chronic":ti,ab,kw,de<br>#5 "Gastritis, Atrophic"[Mesh]<br>#6 "Gastritis, Atrophic":ti,ab,kw,de OR "autoimmune gastritis":ti,ab,kw,de OR ...<br>#7 #1 OR #2 OR #3 OR #4 OR #5 OR #6<br>#8 "rebamipide" [Supplementary Concept]<br>#9 "rebamipide":ti,ab,kw,de OR "OPC-12759":ti,ab,kw,de OR "mucosta":ti,ab,kw,de OR "proamipide":ti,ab,kw,de OR "pramipide":ti,ab,kw,de OR "Recomid":ti,ab,kw,de OR "MCT-SR":ti,ab,kw,de OR "AD-203":ti,ab,kw,de OR synonyms<br>#10 #8 OR #9<br>#11 #7 AND #10<br>#12 #11 AND (meta-analysis OR randomized controlled trial OR systematic review filters)
EMBASE	#1 "gastritis"/exp OR "erosive gastritis"/exp OR "chronic gastritis"/exp<br>#2 "Gastritis":ti,ab,kw,de OR "Gastritides":ti,ab,kw,de OR ... OR "Acute gastritis":ti,ab,kw,de<br>#3 "erosive gastritis":ti,ab,kw,de OR "gastritis, erosive":ti,ab,kw,de<br>#4 "chronic gastritis":ti,ab,kw,de OR "gastritis, chronic":ti,ab,kw,de<br>#5 "atrophic gastritis"/exp<br>#6 "Gastritis, Atrophic":ti,ab,kw,de OR "autoimmune gastritis":ti,ab,kw,de OR ...<br>#7 #1 OR #2 OR #3 OR #4 OR #5 OR #6<br>#8 "rebamipide"/exp<br>#9 "rebamipide":ti,ab,kw,de OR "OPC-12759":ti,ab,kw,de OR "mucosta":ti,ab,kw,de OR "proamipide":ti,ab,kw,de OR "pramipide":ti,ab,kw,de OR synonyms<br>#10 #8 OR #9<br>#11 #7 AND #10<br>#12 #11 AND [randomized controlled trial]/lim
Cochrane Library	#1 [mh "Gastritis"]<br>#2 "Gastritis":ti,ab,kw OR "gastric inflammation":ti,ab,kw OR ... OR "Acute gastritis":ti,ab,kw<br>#3 "erosive gastritis":ti,ab,kw OR "gastritis, erosive":ti,ab,kw

	#4 "chronic gastritis":ti,ab,kw OR "gastritis, chronic":ti,ab,kw<br>#5 [mh "Gastritis, Atrophic"]<br>#6 "Gastritis, Atrophic":ti,ab,kw OR "autoimmune gastritis":ti,ab,kw OR ...<br>#7 #1 OR #2 OR #3 OR #4 OR #5 OR #6<br>#8 "rebamipide":ti,ab,kw OR "OPC-12759":ti,ab,kw OR "mucosta":ti,ab,kw OR "proamipide":ti,ab,kw OR "pramipide":ti,ab,kw OR synonyms<br>#9 #7 AND #8<br>#10 #9 / Trials
ClinicalTrials.gov	"Gastritis" OR "Gastritis, Atrophic" | "rebamipide" OR "OPC-12759" OR "proamipide" OR "pramipide" OR "Mucosta" OR "Mucosta SR" OR "Recomid" OR "Recomid SR" OR "MCT-SR" OR "AD-203
ICTRP	(Gastritis OR "Atrophic Gastritis") AND ("rebamipide" OR "OPC-12759" OR "proamipide" OR "pramipide" OR "Mucosta" OR "Mucosta SR" OR "Recomid" OR "Recomid SR" OR "MCT-SR" OR "AD-203")
RISS	(Gastritis OR 위염) AND (rebamipide OR mucosta OR AD-203 OR 레바미피드 OR 무코스타)
KoreaMed	(Gastritis[TIAB] OR 위염[TIAB]) AND (rebamipide[TIAB] OR mucosta[TIAB] OR "opc 12759"[TIAB] OR proamipide[TIAB] OR pramipide[TIAB] OR Recomid[TIAB] OR "MCT-SR"[TIAB] OR "AD-203"[TIAB])

RISS: Research Information Sharing Service), KoreaMed: Korean medical literature database, ICTRP: International Clinical Trials Registry Platform
Those tables provide the complete search strategies used for each database.

Supplementary Table S2. PRISMA 2020 checklist.

Section and Topic	Item #	Checklist item	Location where item is reported
**TITLE**			
Title	1	Identify the report as a systematic review.	Title: “systematic review and network meta-analysis”
**ABSTRACT**			
Abstract	2	See the PRISMA 2020 for Abstracts checklist.	Abstract, Supplementary table S3
**INTRODUCTION**			
Rationale	3	Describe the rationale for the review in the context of existing knowledge.	1.Introduction: line 45-80
Objectives	4	Provide an explicit statement of the objective(s) or question(s) the review addresses.	1.Introduction: line 82-87
**METHODS**			
Eligibility criteria	5	Specify the inclusion and exclusion criteria for the review and how studies were grouped for the syntheses.	2.3.Eligibility criteria: line 137-141<br>2.1.Outcomes: line 98-110<br>2.5.Data extraction and quality assessment: line 149-152
Information sources	6	Specify all databases, registers, websites, organisations, reference lists and other sources searched or consulted to identify studies. Specify the date when each source was last searched or consulted.	2.2.Data collection methods: line 114-123
Search strategy	7	Present the full search strategies for all databases, registers and websites, including any filters and limits used.	Supplementary TableS1
Selection process	8	Specify the methods used to decide whether a study met the inclusion criteria of the review, including how many reviewers screened each record and each report retrieved, whether they worked independently, and if applicable, details of automation tools used in the process.	2.4.Study selection: line 143-147. Figure 1.
Data collection process	9	Specify the methods used to collect data from reports, including how many reviewers collected data from each report, whether they worked independently, any processes for obtaining or confirming data from study investigators, and if applicable, details of automation tools used in the process.	2.2.Data collection methods: line 116-131<br>2.4.Study selection: line 143-147

Section and Topic	Item #	Checklist item	Location where item is reported
Data items	10a	List and define all outcomes for which data were sought. Specify whether all results that were compatible with each outcome domain in each study were sought (e.g. for all measures, time points, analyses), and if not, the methods used to decide which results to collect.	2.1.Outcomes: line 98-112
	10b	List and define all other variables for which data were sought (e.g. participant and intervention characteristics, funding sources). Describe any assumptions made about any missing or unclear information.	2.5.Data extraction and quality assessment: line 149-152<br>2.7.Statistical analysis: 188-191<br>Page 15: line 425
Study risk of bias assessment	11	Specify the methods used to assess risk of bias in the included studies, including details of the tool(s) used, how many reviewers assessed each study and whether they worked independently, and if applicable, details of automation tools used in the process.	2.5.Data extraction and quality assessment: line 152-156
Effect measures	12	Specify for each outcome the effect measure(s) (e.g. risk ratio, mean difference) used in the synthesis or presentation of results.	2.7.Statistical analysis: line 172-173
Synthesis methods	13a	Describe the processes used to decide which studies were eligible for each synthesis (e.g. tabulating the study intervention characteristics and comparing against the planned groups for each synthesis (item #5)).	2.3.Eligibility criteria: line 137-141<br>2.1.Outcomes: line 98-110<br>2.5.Data extraction and quality assessment: line 149-152
	13b	Describe any methods required to prepare the data for presentation or synthesis, such as handling of missing summary statistics, or data conversions.	2.7.Statistical analysis: line 188-191
	13c	Describe any methods used to tabulate or visually display results of individual studies and syntheses.	Figure 2: Network plots<br>Supplementary Figure S1, S2: Forest plot<br>Supplementary Figure S3: Funnel plot
	13d	Describe any methods used to synthesize results and provide a rationale for the choice(s). If meta-analysis was performed, describe the model(s), method(s) to identify the presence and extent of statistical heterogeneity, and software package(s) used.	2.7.Statistical analysis: line 163-172, line 178-181, line 197-200

Section and Topic	Item #	Checklist item	Location where item is reported
	13e	Describe any methods used to explore possible causes of heterogeneity among study results (e.g. subgroup analysis, meta-regression).	2.7.Statistical analysis: line 167-170, line 187-189
	13f	Describe any sensitivity analyses conducted to assess robustness of the synthesized results.	2.7.Statistical analysis: line 183-189
Reporting bias assessment	14	Describe any methods used to assess risk of bias due to missing results in a synthesis (arising from reporting biases).	Supplementary Figure S3: Funnel plot
Certainty assessment	15	Describe any methods used to assess certainty (or confidence) in the body of evidence for an outcome.	2.6.Grading of the certainty of evidence: line 158-161
**RESULTS**			
Study selection	16a	Describe the results of the search and selection process, from the number of records identified in the search to the number of studies included in the review, ideally using a flow diagram.	3.1.Study selection and characteristics: line 203-205, Figure 1
	16b	Cite studies that might appear to meet the inclusion criteria, but which were excluded, and explain why they were excluded.	3.1.Study selection and characteristics: line 203-205, line 231-235. Figure 1
Study characteristics	17	Cite each included study and present its characteristics.	3.1.Study selection and characteristics: line 206-211, line 226-229. Table 1
Risk of bias in studies	18	Present assessments of risk of bias for each included study.	3.5.Assumptions of NMA and study quality: line 332-333, Figure 3
Results of individual studies	19	For all outcomes, present, for each study: (a) summary statistics for each group (where appropriate) and (b) an effect estimate and its precision (e.g. confidence/credible interval), ideally using structured tables or plots.	3.1.Study selection and characteristics: Table 1
Results of syntheses	20a	For each synthesis, briefly summarise the characteristics and risk of bias among contributing studies.	3.5.Assumptions of NMA and study quality: line 331-338
	20b	Present results of all statistical syntheses conducted. If meta-analysis was done, present for each the summary estimate and its precision	3.2.Efficacy

Section and Topic	Item #	Checklist item	Location where item is reported
		(e.g. confidence/credible interval) and measures of statistical heterogeneity. If comparing groups, describe the direction of the effect.	outcomes: 239-299<br>3.3.Safety outcomes: line 301-315
	20c	Present results of all investigations of possible causes of heterogeneity among study results.	3.4.Sensitivity analysis: line 317-327
	20d	Present results of all sensitivity analyses conducted to assess the robustness of the synthesized results.	3.4.Sensitivity analysis: line 317-327
Reporting biases	21	Present assessments of risk of bias due to missing results (arising from reporting biases) for each synthesis assessed.	3.5.Assumptions of NMA and study quality: line 331-337. Figure 3
Certainty of evidence	22	Present assessments of certainty (or confidence) in the body of evidence for each outcome assessed.	3.6.GRADE assessment: line 342-351. Supplementary Table S6
**DISCUSSION**			
Discussion	23a	Provide a general interpretation of the results in the context of other evidence.	4.Discussion: line 362-364, line 379-383
	23b	Discuss any limitations of the evidence included in the review.	4.Discussion: line 397-406
	23c	Discuss any limitations of the review processes used.	4.Discussion: line 410-404
	23d	Discuss implications of the results for practice, policy, and future research.	4.Discussion: line 484-488, line 397-404
**OTHER INFORMATION**			
Registration and protocol	24a	Provide registration information for the review, including register name and registration number, or state that the review was not registered.	2.Materials and Methods: line 95-96
	24b	Indicate where the review protocol can be accessed, or state that a protocol was not prepared.	2.Materials and Methods: line 96
	24c	Describe and explain any amendments to information provided at registration or in the protocol.	No amendments
Support	25	Describe sources of financial or non-financial support for the review, and the role of the funders or sponsors in the review.	Page 15: line 425,<br>Page 16: line 431-434
Competing interests	26	Declare any competing interests of review authors.	Page 16: line 431

Section and Topic	Item #	Checklist item	Location where item is reported
Availability of data, code and other materials	27	Report which of the following are publicly available and where they can be found: template data collection forms; data extracted from included studies; data used for all analyses; analytic code; any other materials used in the review.	Page 165 line 428-429

Supplementary Table S3. PRISMA 2020 abstract checklist

Section and Topic	Item #	Checklist item	Reported (Yes/No)
**TITLE**			
Title	1	Identify the report as a systematic review.	Yes
**BACKGROUND**			
Objectives	2	Provide an explicit statement of the main objective(s) or question(s) the review addresses.	Yes
**METHODS**			
Eligibility criteria	3	Specify the inclusion and exclusion criteria for the review.	Yes
Information sources	4	Specify the information sources (e.g. databases, registers) used to identify studies and the date when each was last searched.	Yes
Risk of bias	5	Specify the methods used to assess risk of bias in the included studies.	Yes
Synthesis of results	6	Specify the methods used to present and synthesise results.	Yes
**RESULTS**			
Included studies	7	Give the total number of included studies and participants and summarise relevant characteristics of studies.	Yes
Synthesis of results	8	Present results for main outcomes, preferably indicating the number of included studies and participants for each. If meta-analysis was done, report the summary estimate and confidence/credible interval. If comparing groups, indicate the direction of the effect (i.e. which group is favoured).	Yes
**DISCUSSION**			
Limitations of evidence	9	Provide a brief summary of the limitations of the evidence included in the review (e.g. study risk of bias, inconsistency and imprecision).	No<br>(Limitation not reported in the abstract.)
Interpretation	10	Provide a general interpretation of the results and important implications.	Yes
**OTHER**			
Funding	11	Specify the primary source of funding for the review.	No<br>(Funding not reported in the abstract)
Registration	12	Provide the register name and registration number.	No<br>(PROSPERO registration (CRD420251127024) is reported in Methods, not in abstract.)

Supplementary Table S4. Inclusion and exclusion criteria of included studies.

Study ID	Inclusion Criteria	Exclusion Criteria
**HW Han et al., 2011**	Patients aged 19-65 years<br>Patients diagnosed with acute or chronic gastritis<br>Patients with one or more distinct mucosal erosions in the gastric mucosa, confirmed by endoscopy	Patients with peptic ulcers (excluding scarred lesions) or reflux esophagitis<br>Patients with a history of malignancy<br>Patients with a history of anti-acid secretory surgery or surgery involving the stomach or esophagus (excluding simple perforation repair)<br>Patients with abnormal liver or kidney function, defined as serum AST, ALT, or creatinine levels exceeding three times the upper limit of normal<br>Patients with severe hepatic, renal, cardiac, pulmonary, hematologic, or other disorders that may affect the clinical trial<br>Patients with Zollinger-Ellison syndrome<br>Patients known to be hypersensitive to the investigational product or its components.<br>Patients who have received PPIs, APAs, H2RAs, muscarinic receptor antagonists, or mucosal protective agents within 2 weeks before enrollment<br>Patients who require continuous use of medications that may affect the trial, such as NSAIDs, acetaminophen, or corticosteroids<br>Patients with psychiatric disorders or those with a history of drug or alcohol abuse<br>Pregnant or breastfeeding women<br>Any individual deemed by the investigator to be unsuitable for participation in the trial.
**Unpublished, 1999**	Patients aged 18-75 years<br>Patients diagnosed with acute or chronic gastritis based on endoscopic examination.<br>Patients with endoscopic evidence of gastric mucosal lesions such as erosions, bleeding, erythema, or edema<br>Patients with subjective or objective symptoms requiring medical treatment	Patients with peptic ulcers (excluding scarred lesions) or reflux esophagitis<br>Patients who have received H2RAs, muscarinic receptor antagonists, or NSAIDs within 2 weeks before the start of the study<br>Patients who are currently taking mucosal protective agents at the time of study initiation<br>Patients who are undergoing maintenance therapy at study initiation and have experienced recurrence<br>Patients with a history of gastrectomy<br>Patients with severe hepatic, renal, cardiac, pulmonary, hematologic, or other disorders<br>Patients with complications of duodenal ulcer<br>Patients with malignant tumors of the gastrointestinal tract<br>Patients taking medications considered likely to affect the outcome of the study<br>Women who are pregnant, possibly pregnant, suspected of being pregnant, or breastfeeding<br>Any other individuals deemed inappropriate for participation by the investigator
**SY Seol et al., 2004**	Patients with erosive gastritis diagnosed by endoscopy	Patients with peptic ulcer disease and reflux esophagitis<br>Patients with the presence of a malignant tumor in the digestive tract<br>Thrombotic patients (cerebral thrombosis, myocardial infarction, thrombophlebitis, etc.)<br>Patients with the presence of significant hematological, renal, cardiac, pulmonary, or hepatic abnormalities<br>Patients using drugs capable of interfering with digestive mucosal integrity, gastric secretion, or gastrointestinal motility, including H2RAs, NSAIDs, muscarinic antagonists, and gastroprotective agents (within the previous 14 days)<br>Patients with consumption coagulopathy<br>Patients with a history of hypersensitivity to drugs
**YJ Choi et al., 2017**	Patients aged 20-75 years<br>Patients diagnosed with acute or chronic gastritis<br>Patients with baseline endoscopic findings with one or more erosions	Patients with peptic ulcer or gastroesophageal reflux disease<br>Patients who had undergone a previous GI operation, such as an operation to inhibit gastric acid secretion or gastrectomy (simple stomach perforation operation was excluded)<br>Patients with significant impairments in the hematologic, renal, cardiac, pulmonary, hematopoietic, and endocrine systems<br>Patients who used any prokinetics, H2RAs, PPIs, anticholinergic drugs (muscarinic receptor antagonists), gastrin receptor antagonists, protective factor enhancers, gastric mucosal protective agents, or NSAIDs within 2 weeks of the screening test<br>Patients with known hypersensitivity to the investigational drug<br>Women who were pregnant or lactating<br>Women of childbearing age who are not using contraception

**SY Seo et al., 2023**	Patients aged >=19 years<br>Patients diagnosed with acute or chronic gastritis<br>Patients with one or more gastric erosions on baseline esophagogastroduodenoscopy (EGD)<br>Patients who had gastrointestinal symptoms at the time of enrollment or within 7 days	Patients with peptic ulcer or reflux esophagitis<br>Patients who had undergone gastrointestinal surgery, such as surgery to inhibit gastric acid secretion or esophagogastric surgery (except simple closure of peptic ulcer perforation)<br>Patients with a history of gastrointestinal malignancy<br>Patients with Zollinger-Ellison syndrome<br>Patients with a history of thrombotic disorder (cerebral infarction, myocardial infarction, thrombophlebitis) or coagulation disorder<br>Patients with abnormal serum creatinine (>1.5 times ULN)<br>Patients with abnormal aspartate aminotransferase or alanine aminotransferase (>2 times the upper limit of normal)<br>Patients with genetic disorders such as galactose intolerance, Lactase deficiency, or glucose-galactose malabsorption<br>Patients who had used antithrombotic agents such as warfarin<br>Patients who took any of the drugs of H2RAs, PPIs, antacids, potassium competitive acid blockers, prokinetics, prostaglandin analogs, gastric mucosal protective agents, corticosteroids, NSAIDs, or aspirin within 2 weeks before enrollment<br>Patients with known hypersensitivity to the investigational drugs<br>Patients who took any other clinical trial medications within 3 months before enrollment<br>Pregnant or lactating patients<br>Fertile women not using medically permitted contraceptive methods<br>Patients who were unable to undergo EGD<br>Patients with any other conditions or diseases that were regarded as unsuitable by the investigator
**GH Kim et al., 2021**	Patients aged 20-75 years<br>Patients diagnosed with acute or chronic gastritis<br>Patients with one or more gastric erosions on baseline EGD	Patients with peptic ulcer or reflux esophagitis<br>Patients who had undergone gastrointestinal surgery, such as an operation to inhibit gastric acid secretion and esophagogastric surgery<br>Patients with a history of gastrointestinal malignancy<br>Patients with significant impairments in the hematologic, renal, cardiac, pulmonary, hematopoietic, and endocrine systems<br>Patient who had used any H2RAs, PPIs, gastrin receptor antagonists, anticholinergic drugs, prokinetics, prostaglandin analogs, or gastric mucosal protective agents within 2 weeks of the investigational drug administration<br>Patients who should take corticosteroids, NSAIDs, aspirin, or antithrombotic agents during the study period<br>Patients with known hypersensitivity to rebamipide<br>Women who were pregnant or lactating<br>Women of childbearing age who are not using contraception
**SH Park et al., 2025**	Patients aged >19 years<br>Patients with one or more gastric erosions on baseline EGD at the time of enrollment or within 7 days before enrollment<br>Patients who had gastrointestinal symptoms	Patients with a history of peptic ulcer or reflux esophagitis<br>Patients who had undergone gastrointestinal surgery except for simple closure of a peptic ulcer perforation<br>Patients with a history of gastrointestinal malignancy<br>Patients with Zollinger-Ellison syndrome<br>Patients with a history of thrombotic disorder or coagulation disorder<br>Patients with abnormal alanine aminotransferase, alkaline phosphatase, or serum creatinine (>3 times the upper limits of normal levels)<br>Patients with genetic disorders (e.g., galactose intolerance, Lactase deficiency, glucose-galactose malabsorption)<br>Patients who use antithrombotic agents during the study period<br>Patients who had used any H2RAs, PPIs, antacids, prokinetics, prostaglandin analogs, or gastric mucosal protective agents within 2 weeks of the study drug administration<br>Patients who were required to take corticosteroids, NSAIDs, or aspirin during the study period<br>Patients with known hypersensitivities to the study drug<br>Patients taking any other clinical trial medications within 28 days before the study start date<br>Patients who were pregnant or lactating<br>Fertile women who did not consent to use medically permitted contraceptive methods<br>Patients with any other conditions or diseases that were regarded as unsuitable by the investigator<br>Patients who were unavailable for upper endoscopy

**JJ Jeong et a l., 2007**	Patients aged 18-75 years<br>Patients diagnosed with acute or chr onic gastritis<br>Patients with endoscopically confirm ed gastric mucosal erosions (elevated or flat) showing mucosal defects<br>Patients presented with subjective or objective symptoms requiring medica l treatment	Patients with peptic ulcers (excluding scarred lesions) or reflux esopha gitis<br>Patients with malignant tumors of the gastrointestinal tract<br>Patients with a history of gastrectomy<br>Patients with severe medical or surgical conditions<br>Patients with abnormal liver or kidney function, defined as AST, ALT, or creatinine levels >3 times the upper limit of normal<br>Patients with known hypersensitivity to the investigational drug<br>Patients taking medications that may affect the outcome of the study<br>Pregnant or breastfeeding women<br>Patients who are expected to have poor compliance with medication or endoscopic procedures.<br>Patients deemed by the investigator to be inappropriate for participatio n in the study.
**Unpublished, 2 014**	Patients aged 20-75 years<br>Patients diagnosed with acute or chr onic gastritis based on upper endosc opy.<br>Patients with one or more erosions i dentified on upper endoscopy (active erosion showing mucosal defect wit h whitish exudate or bleeding, exclu ding verrucous erosions)	Patients with peptic ulcers (excluding scarred lesions) or gastroesophag eal reflux<br>Patients with a current or past history of gastrointestinal malignancy, o r a history of malignancy in other organs within the past 5 years<br>Patients with a history of gastroesophageal surgery, including acid-supp ressive surgery or gastrectomy (excluding simple perforation)<br>Patients with Zollinger-Ellison syndrome<br>Patients with a history of thrombosis or those currently taking anticoag ulants such as warfarin<br>Patients with severe disorders of the liver, kidneys, heart, lungs, hemat ologic system, or endocrine system<br>Patients with psychiatric or neurological disorders, alcohol dependence, or drug abuse<br>Patients who have received any of the medications of gastrointestinal motility regulators, H2RAs, PPIs, anticholinergics, gastrin receptor anta gonists, protective factor enhancers, gastric mucosal protectors, or NSA IDs within 2 weeks before study initiation<br>Patients with a known hypersensitivity to the investigational drug<br>Patients taking medications that may affect the outcome of the study<br>Women who are pregnant, suspected of being pregnant, or breastfeedin g<br>Any individual deemed by the investigator to be unsuitable for particip ation in the study
**JH Kim et al., 2005**	Patients diagnosed with acute or chr onic gastritis based on upper endosc opy, revealing gastric mucosal lesion s (e.g., erosion, bleeding, erythema, or edema)<br>Patients with gastrointestinal sympto ms requiring medical treatment	Patients with peptic ulcers (excluding scarred lesions) or reflux esopha gitis<br>Patients with gastrointestinal malignancies or a history of gastrectomy<br>Patients with thrombosis (cerebral thrombosis, coronary thrombosis, thr ombotic phlebitis)<br>Patients with severe medical or surgical conditions<br>Patients with consumptive coagulopathies<br>Patients with a known history of hypersensitivity to the investigational drug<br>Patients taking medications that may affect the outcome of the study<br>Pregnant or breastfeeding women<br>Patients expected to have poor compliance with medication or endosco pic procedures.
**JS Moon et a l., 2013**	Patients aged 20-75 years<br>Patients with erosions endoscopically confirmed<br>Patients with gastric symptoms requi ring medical treatment	Patients with active or healing-stage peptic ulcers (excluding scarred le sions) or reflux esophagitis<br>Patients with a history of malignancy<br>Patients with a history of acid-suppressive surgery or surgery involving the stomach or esophagus<br>Patients with genetic disorders (galactose intolerance, lactase deficienc y, or glucose-galactose malabsorption)<br>Patients with severe liver, kidney, cardiovascular, gastrointestinal, respi ratory, endocrine, or central nervous system diseases<br>Patients with thrombosis or consumptive coagulopathy<br>Patients with psychiatric illness or those currently taking medications t hat could affect the study outcome<br>Patients with AST, ALT, total bilirubin, or creatinine levels that are no t less than 2 times the upper limit of normal<br>Patients who have taken any medications of H2RAs, PPIs, anticholiner gics, gastrin receptor antagonist, prostaglandin analogs, or other gastriti s medications within 2 weeks before administration of the investigation al drug<br>Patients expected to use NSAIDs, corticosteroids, or aspirin during the study period (except those who continuously have taken aspirin no mor e than 100mg, corticosteroid, or clopidogrel)<br>Patients with a known history of hypersensitivity to the investigational drug<br>Patients who have received another investigational drug within 30 days before enrollment

	Women who are pregnant, breastfeeding, or of childbearing age withou t contraception<br>Any other individuals deemed by the investigator to be unsuitable for t he study participation due to factors that may affect the study outcom e.

APAs: Acid-pump antagonists, H2RAs: Histamine H2 receptor antagonists, NSAIDs: Nonsteroidal anti-inflammatory drugs, PPIs: Proton pump inhibitors.

Supplementary Table S5. Sensitivity analysis.

Double-blind studies

Outcome	Treatment duration	Analysis set	OR	95% CI for OR
**Improvement rate**	2 weeks	FAS	1.11	(0.88, 1.39) S_NE)
**Improvement rate**	2 weeks	PPS	1.09	(0.85, 1.41) S_NE)
**Improvement rate**	2, 3, 4 weeks	FAS	1.02	(0.89, 1.18) S_NE)
**Improvement rate**	2, 3, 4 weeks	PPS	1.02	(0.87, 1.19) S_NE)
**Cure rate**	2 weeks	FAS	1.03	(0.84, 1.26) NS_D)
**Cure rate**	2 weeks	PPS	1.00	(0.81, 1.24) NS_D)
**Cure rate**	2, 3, 4 weeks	FAS	0.98	(0.87, 1.10) NS_D)
**Cure rate**	2, 3, 4 weeks	PPS	0.98	(0.87, 1.10) NS_D)

Studies with low risk of bias

Outcome	Treatment duration	Analysis set	OR	95% CI for OR
**Improvement rate**	2 weeks	FAS	1.06	(0.88, 1.28) S_NE)
**Improvement rate**	2 weeks	PPS	1.04	(0.86, 1.27) S_NE)
**Improvement rate**	2, 3, 4 weeks	FAS	0.99	(0.90, 1.10) S_NE)
**Improvement rate**	2, 3, 4 weeks	PPS	0.98	(0.88, 1.10) S_NE)
**Cure rate**	2 weeks	FAS	1.03	(0.84, 1.26) NS_D)
**Cure rate**	2 weeks	PPS	1.00	(0.81, 1.24) NS_D)
**Cure rate**	2, 3, 4 weeks	FAS	0.98	(0.89, 1.09) NS_D)
**Cure rate**	2, 3, 4 weeks	PPS	0.98	(0.87, 1.10) NS_D)

Published studies

Outcome	Treatment duration	Analysis set	OR	95% CI for OR
**Improvement rate**	2 weeks	FAS	1.06	(0.88, 1.28) S_NE)
**Improvement rate**	2 weeks	PPS	1.04	(0.86, 1.27) S_NE)
**Improvement rate**	2, 3, 4 weeks	FAS	0.97	(0.84, 1.11) S_NE)
**Improvement rate**	2, 3, 4 weeks	PPS	0.96	(0.83, 1.11) S_NE)
**Cure rate**	2 weeks	FAS	1.03	(0.84, 1.26) NS_D)
**Cure rate**	2 weeks	PPS	1.00	(0.81, 1.24) NS_D)
**Cure rate**	2, 3, 4 weeks	FAS	0.95	(0.83, 1.09) NS_D)
**Cure rate**	2, 3, 4 weeks	PPS	0.96	(0.82, 1.11) NS_D)

OR: Odds ratio of Stillen® comparing to rebamipide, CI: Confidence interval

FAS: Full analysis set, PPS: Per-protocol set

S_NE) Significance for non-inferiority of Stillen® comparing to rebamipide at one-sided 2.5% significance level (non-inferiority margin = 0.56)

S_NE) No significance for the difference between Stillen® and rebamipide at a two-sided 5% significance level

Supplementary Table S6. Grade assessment.

Certainty assessment							№ of patients		Effect		Certainty	Importance
**№ of studies**	**Study design**	**Risk of bias**	**Inconsistency**	**Indirectness**	**Imprecision**	**Other considerations**	**Stillen®**	**Rebamipide**	**Relative (95%CI)**	**Absolute (95% CI)**		
**Improvement rate at 2 weeks (FAS) (follow-up: range 2 weeks to 2 weeks; assessed with: Endoscopy)**												
**3**	randomised trials	not serious	not serious	not serious	not serious	none	93/209 (44.5%)	20/42 (47.6%)	**OR 1.11** (0.88 to 1.39)	**26 more per 1,000** (from 32 fewer to 82 more)	⊕⊕⊕⊕ High	CRITICAL
**Improvement rate at 2 weeks (PPS) (follow-up: range 2 weeks to 2 weeks; assessed with: Endoscopy)**												
**3**	randomised trials	not serious	not serious	not serious	not serious	none	83/189 (43.9%)	19/38 (50.0%)	**OR 1.09** (0.85 to 1.41)	**22 more per 1,000** (from 41 fewer to 85 more)	⊕⊕⊕⊕ High	CRITICAL
**Improvement rate at 2, 3, 4 weeks (FAS) (follow-up: range 2 weeks to 4 weeks; assessed with: Endoscopy)**												
**9**	randomised trials	not serious	not serious	not serious	not serious	none	344/718 (47.9%)	169/304 (55.6%)	**OR 0.99** (0.88 to 1.10)	**2 fewer per 1,000** (from 32 fewer to 23 more)	⊕⊕⊕⊕ High	CRITICAL
**Improvement rate at 2, 3, 4 weeks (PPS) (follow-up: range 2 weeks to 4 weeks; assessed with: Endoscopy)**												
**9**	randomised trials	not serious	not serious	not serious	not serious	none	314/627 (50.1%)	149/264 (56.4%)	**OR 0.98** (0.87 to 1.11)	**5 fewer per 1,000** (from 34 fewer to 25 more)	⊕⊕⊕⊕ High	CRITICAL
**Cure rate at 2 weeks (FAS) (follow-up: range 2 weeks to 2 weeks; assessed with: Endoscopy)**												
**1**	randomised trials	not serious	not serious	not serious	serious	none	21/48 (43.8%)	17/42 (40.5%)	**OR 1.03** (0.84 to 1.26)	**7 more per 1,000** (from 41 fewer to 57 more)	⊕⊕⊕◯ Moderate	IMPORTANT
**Cure rate at 2 weeks (PPS) (follow-up: range 2 weeks to 2 weeks; assessed with: Endoscopy)**												
**1**	randomised trials	not serious	not serious	not serious	serious	none	18/43 (41.9%)	16/38 (42.1%)	**OR 1.00** (0.81 to 1.24)	**0 fewer per 1,000** (from 50 fewer to 53 more)	⊕⊕⊕◯ Moderate	IMPORTANT
**Cure rate at 2, 3, 4 weeks (FAS) (follow-up: range 2 weeks to 4 weeks; assessed with: Endoscopy)**												
**7**	randomised trials	not serious	not serious	not serious	not serious	none	231/557 (41.5%)	144/304 (47.4%)	**OR 0.96** (0.88 to 1.05)	**10 fewer per 1,000** (from 32 fewer to 12 more)	⊕⊕⊕⊕ High	IMPORTANT
**Cure rate at 2, 3, 4 weeks (PPS) (follow-up: range 2 weeks to 4 weeks; assessed with: Endoscopy)**												
**7**	randomised trials	not serious	not serious	not serious	not serious	none	215/481 (44.7%)	123/264 (46.6%)	**OR 0.96** (0.87 to 1.06)	**10 fewer per 1,000** (from 34 fewer to 15 more)	⊕⊕⊕⊕ High	IMPORTANT

FAS: Full analysis set, PPS: Per-protocol set, OR: Odds ratio, CI: Confidence interval

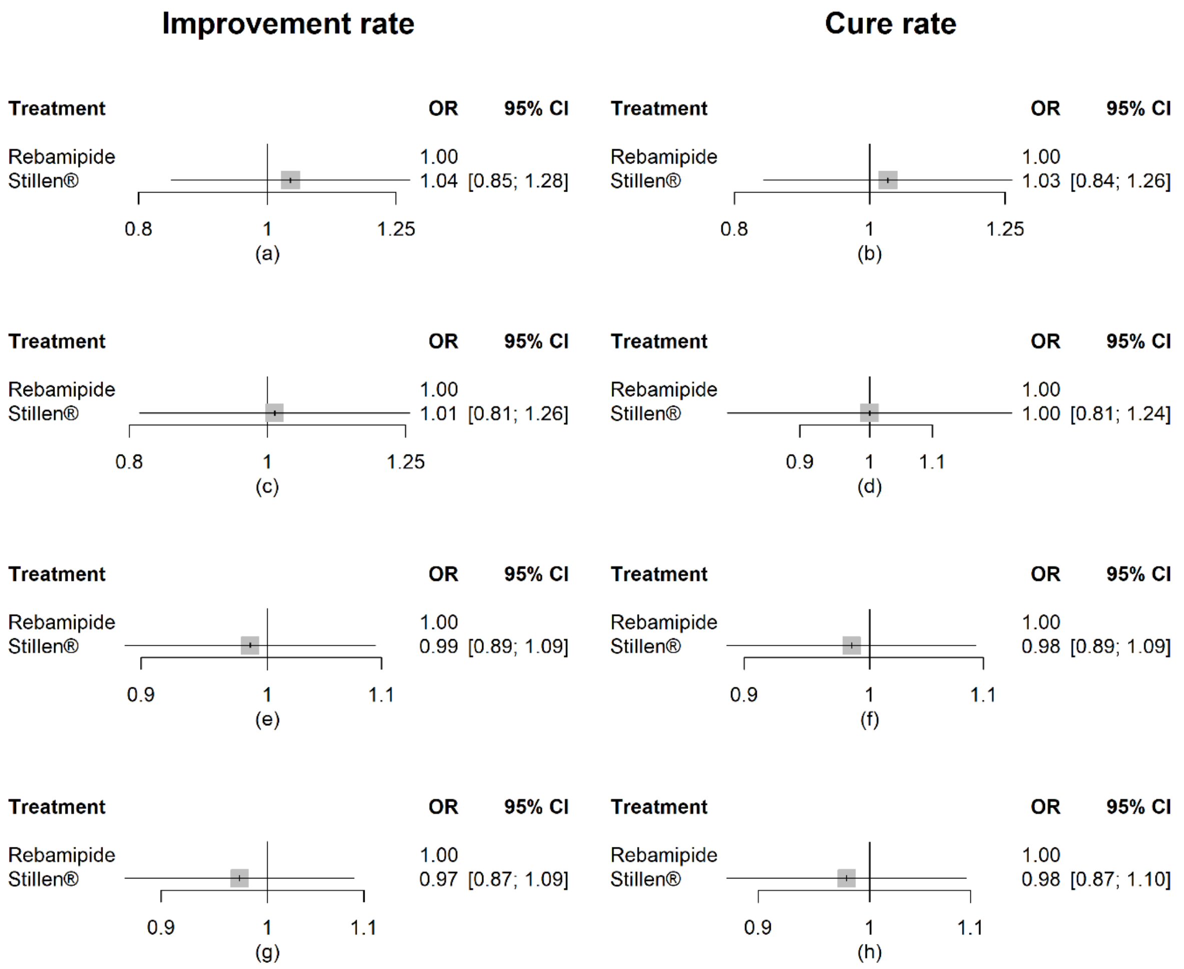

Supplementary Figure S1. Forest plot: Direct comparisons in efficacy outcomes. (a), (b): 2 weeks (FAS). (c), (d): 2weeks (PPS). (e), (f): 2, 3, 4 weeks (FAS). (g), (h): 2, 3, 4 weeks (PPS). FAS: Full analysis set, PPS: Per-protocol set, OR: Odds ratio of Stillen® compared to rebamipide, CI: Confidence interval.

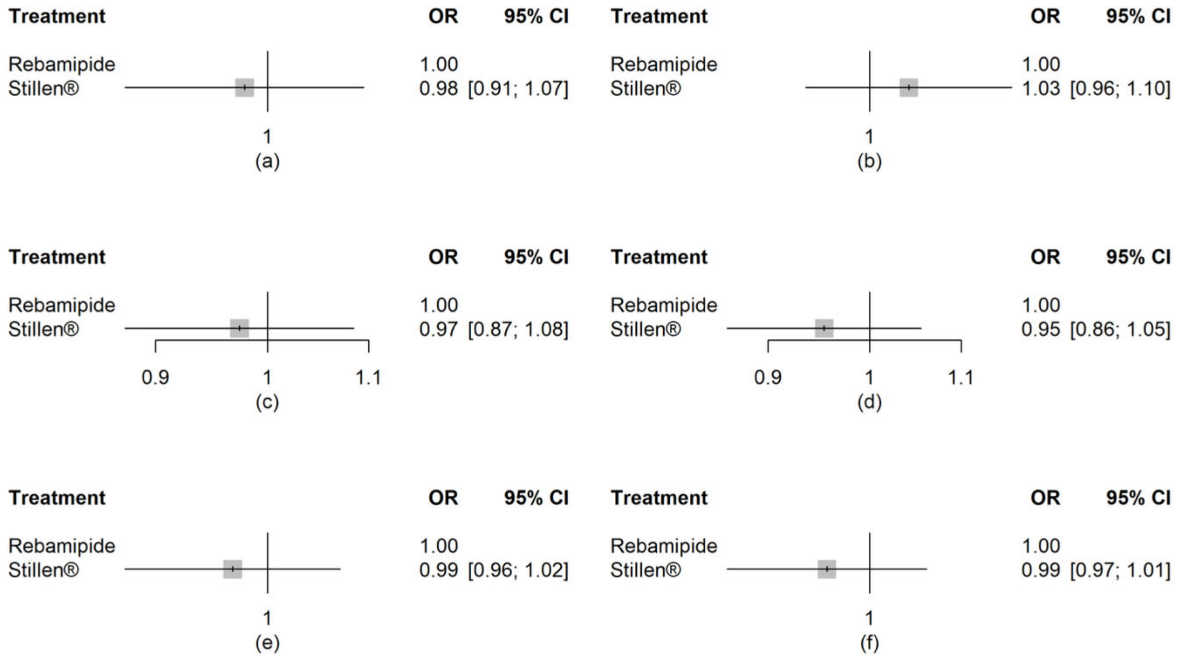

Supplementary Figure S2. Forst plot: Direct comparisons in safety outcomes. (a): AE, 2 weeks. (b): GI disorder, 2, 3, 4 weeks. (c): ADR, 2 weeks. (d): GI disorder, 2 weeks. (e): ADR, 2, 3, 4 weeks. (f): GI disorder, 2, 3, 4 weeks. AE: Adverse event, GI: Gagstrointestinal, ADR: Adverse drug reaction. OR: Odds ratio of Stillen® compared to rebamipide, CI: Confidene interval

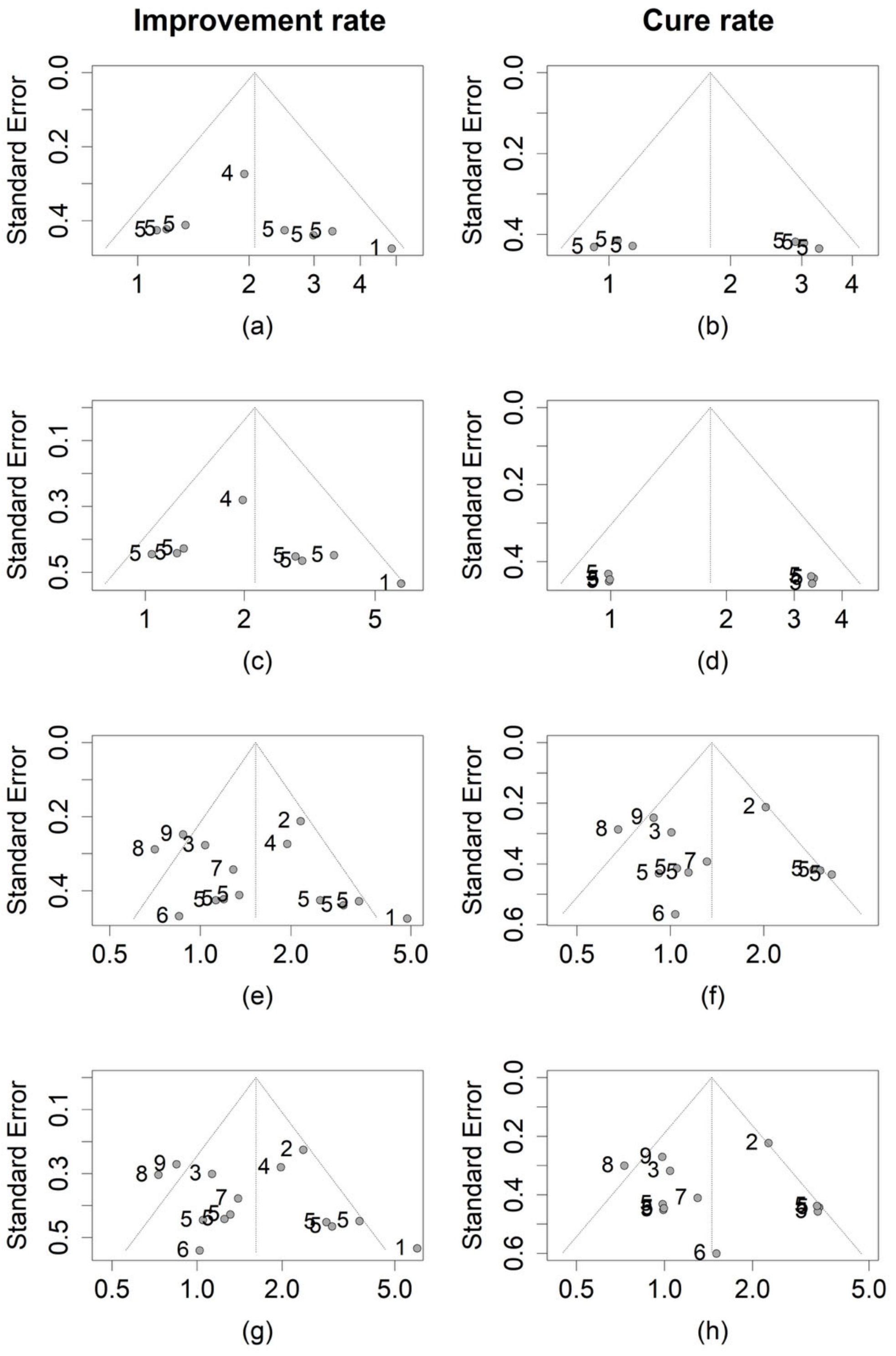

Supplementary Figure S3. Funnel plot. (a), (b): Improvement rate at 2 weeks (n=3, n=1), (c), (d): Improvement rate at 2, 3 and 4 weeks (n=3, n=1), (e), (f): Cure rate at 2 weeks (n=9, n=7), (g), (h): Cure rate at 2, 3, and 4 weeks (n=9, n=7). 1: Unpublished, 1999 [35], 2: SY Seol set al., 2004 [17], 3: HW Han et al., 2011 [21], 4: SY Seo et al., 2023 [26], 5: SH Park et al., 2025 [25], 6: JJ Jeong et al., 2007 [23], 7: JH Kim et al., 2005 [36], 8: JS Moon et al., 2013 [24] , 9: Unpublished, 2014 [32]. (e) and (f) represent single study outcome, and assessment of publication bias using funnel plots was not feasible.
